# Experimental association between diabetes and pregnancy: renal effects for mothers in the postpartum period and possible signaling pathways involved

**DOI:** 10.1186/1758-5996-7-S1-A71

**Published:** 2015-11-11

**Authors:** Nathane França Silva, Natany Garcia Reis, Pâmella Francis dos Santos, Ana Paula Coelho Balbi

**Affiliations:** 1 Universidade Federal de Uberlândia (UFU), Uberlândia, Brazil

## Background

The Renin Angiotensin Aldosterone System, through the Angiotensin II (AII), plays important roles during pregnancy that is associated with marked increases in renal hemodynamics and may be affected by diabetes mellitus (DM). AII binding to its receptors, activates the MAPK (Mitogenic Activated Protein Kinase) cascade.

## Objectives

Evaluate the structural and functional renal changes and the expression of MAPK in the renal cortex of Wistar rats with DM induced during pregnancy and maintained postpartum.

## Materials and methods

There were 4 groups (G): G1 (non-pregnant controls rats), females that received intraperitoneal injection (ipi) of 0.9% saline (s), G2 (non-pregnant diabetic rats), females that received ipi of alloxan (100 mg/kg) diluted in 0.9% s, G3 (control mothers), mothers that received ipi of 0.9% s and G4 (diabetic mothers), mothers that received ipi of alloxan (100 mg/kg) diluted in 0.9% s. The females with blood glucose above 150 mg/dL were considered diabetic. About 50 days after delivery for G3 and G4 and corresponding time for G1 and G2, the rats were subjected to structural and functional renal studies.

## Results

G2 and G4 animals had glycemia and urinary volume higher than control animals. The blood pressure was not different among the groups, but there was a tendency for reduction in glomerular filtration rate from G4 when compared to the other G. There was also a significant increase in kidney weight/body weight ratio of G4 in relation to other G. Morphometric analysis showed renal corpuscle and the capsular space area did not differ between G studied, but there was an augment in glomerular tuft area in G3 and G4. G2 and G4 presented higher percentage of cortical collagen, while the count of mast cells did not differ between G. G3 showed higher glomerular cell proliferation rate when compared to G1 and G2, but in G4 this rate was smaller than G3. On the other hand, in the tubulointerstitial (TBI) compartment cell proliferation was higher in G4. Glomerular and TBI expression of α-SMA (Smooth muscle actin) were increased in G4 compared to other G. G4 showed a reduction of p-p38+ glomerular cells in relation to other G. However, p-JNK expression was higher in both the glomeruli and TBI compartment in G4 compared to other G.

## Conclusion

DM induced during pregnancy and maintained in the postpartum period in Wistar rats resulted in impairment in kidney and these alterations may be related to changes in the expression of p-p38 and p-JNK MAPK.

